# Daily rhythms of the sleep-wake cycle

**DOI:** 10.1186/1880-6805-31-5

**Published:** 2012-03-13

**Authors:** Jim Waterhouse, Yumi Fukuda, Takeshi Morita

**Affiliations:** 1 Research Institute for Sport and Exercise Physiology, Liverpool John Moores University, Liverpool, UK; 2 Department of Living Environmental Science, Fukuoka Women's University, Japan

**Keywords:** Adolescence, chronotype, circadian rhythm, endogenous component, exogenous component, old age, sleep homeostat, time awake

## Abstract

The amount and timing of sleep and sleep architecture (sleep stages) are determined by several factors, important among which are the environment, circadian rhythms and time awake. Separating the roles played by these factors requires specific protocols, including the constant routine and altered sleep-wake schedules. Results from such protocols have led to the discovery of the factors that determine the amounts and distribution of slow wave and rapid eye movement sleep as well as to the development of models to determine the amount and timing of sleep. One successful model postulates two processes. The first is process S, which is due to sleep pressure (and increases with time awake) and is attributed to a 'sleep homeostat'. Process S reverses during slow wave sleep (when it is called process S'). The second is process C, which shows a daily rhythm that is parallel to the rhythm of core temperature. Processes S and C combine approximately additively to determine the times of sleep onset and waking. The model has proved useful in describing normal sleep in adults. Current work aims to identify the detailed nature of processes S and C. The model can also be applied to circumstances when the sleep-wake cycle is different from the norm in some way. These circumstances include: those who are poor sleepers or short sleepers; the role an individual's chronotype (a measure of how the timing of the individual's preferred sleep-wake cycle compares with the average for a population); and changes in the sleep-wake cycle with age, particularly in adolescence and aging, since individuals tend to prefer to go to sleep later during adolescence and earlier in old age. In all circumstances, the evidence that sleep times and architecture are altered and the possible causes of these changes (including altered S, S' and C processes) are examined.

## Review

### Sleep in adults

Most adults take a consolidated 7-hour sleep during the night [1]. The reasons for sleeping at night are partly because the environment is quiet and also it would be unconventional to arrange meetings or meet friends at this time. Moreover, we are diurnal creatures and after a normal day when we have been awake and active for some time, we feel tired in the evening and ready for sleep. It is possible to sleep at other times, as is evident from the lifestyle of night workers but, even in a quiet environment, daytime sleep tends to be more fragmented and shorter than nocturnal sleep. That is, the ability to get to sleep and sleep uninterruptedly for long enough shows a daily rhythm. This rhythm of ease of getting to sleep (sleep propensity) is clear if individuals miss a night's sleep; they feel tired during the night but, in spite of having had no sleep, they then feel less tired as the new day dawns and, during the afternoon, will feel surprisingly alert. However, by the evening, the sensation of fatigue increases markedly and becomes increasingly difficult to resist. This result indicates that there is an increasing drive to sleep as the amount of time awake continues to rise but that it is mixed with a rhythmic component that varies during the course of the 24 hours.

A good sleep is recuperative and removes the feelings of fatigue (and also produces an improvement in cognitive ability); individuals then feel ready to face the rigors of a new day. Intuitively, the concept of increasing 'sleep pressure' with increasing time awake is not difficult to appreciate (even if the detailed nature of sleep pressure is not understood). However, the presence of daily rhythms in the desire to sleep, staying asleep and waking up might be less easy to understand. In fact, when repeated measurements are made over the course of 24 hours in subjects who are living normally (active in the daytime and sleeping at night), all physiological and biochemical variables show daily rhythms. Some properties of such rhythmicity need to be considered, and they will be illustrated by the rhythm of core temperature (which is representative of biological rhythms in general).

### Basic chronobiology

#### Endogenous and exogenous components of a rhythm

**Figure 1:**
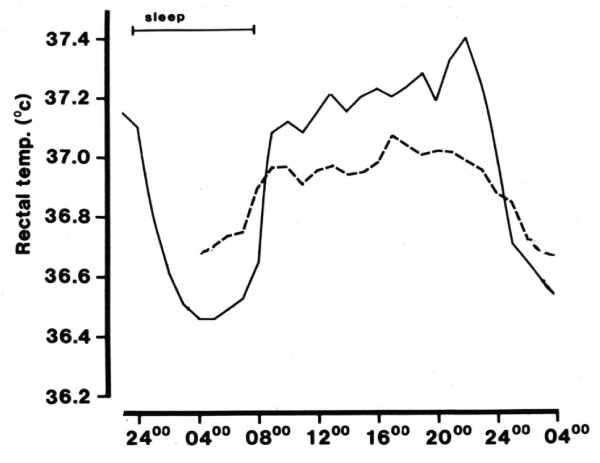
**The daily rhythm of core temperature in a group of eight young men**. Full line: under normal conditions (sleep from 12 a.m. to 7 a.m., indicated by bar). Dashed line: undergoing a 24-hour constant routine starting at 4 a.m. (From [2]).

Figure 1 shows the 24-hour rhythm of core temperature in a group of subjects living normally. The temperature is higher in the daytime and lower at night. *A priori*, it would be supposed that such rhythms result from the behavioral changes that are associated with the sleep-activity cycle. During the daytime, individuals are physically and mentally active, and eat and drink in an environment that is busy and stimulating; all of these factors will tend to raise core temperature. By contrast, at night, individuals fast, are inactive, sleep and choose a quiet, dark environment.

In fact, such an explanation is only partially correct; the standard method for demonstrating this is the 'constant routine'. In this protocol, a subject is required to remain awake and sedentary (or lying down) and relaxed for at least 24 hours in an environment of constant temperature, humidity and lighting; to engage in similar activities, generally reading or listening to music; and to take identical meals at regularly-spaced intervals. Such a protocol removes any rhythmic changes due to the individual's lifestyle or environment. When this protocol is undertaken, it is observed (Figure 1) that the rhythm of core temperature does not disappear, even though its amplitude decreases. The rhythm shows a peak around 4 p.m. to 6 p.m. and a minimum around 4 a.m. to 5 a.m. The rhythm is quite symmetrical, with the time of most rapid rise being in the period 7 a.m. to 10 a.m. and that of most rapid fall around 10 a.m. to 1 a.m.

By considering the two temperature profiles shown in Figure 1, the following deductions can be made [2,3]:

(i) The rhythm observed during the constant routine arises internally; it is the endogenous component of the temperature rhythm and its generation is attributed to a 'body clock'.

(ii) Since the two rhythms are not identical, effects due to the environment and lifestyle are also present when the participants live conventionally; the difference between the two rhythms is the exogenous component of the rhythm. The exogenous component of core temperature is dominated by the sleep-wake cycle, activity raising temperature and lying down and sleeping decreasing it.

(iii) The two components are in phase. During the daytime, body temperature is raised by the body clock acting in synchrony with a more dynamic environment and increased physical and mental activities; during the night, the clock, a more restful environment and relaxed lifestyle all act to reduce core temperature.

These deductions are general, insofar as all rhythms show a mixture of endogenous and exogenous components when compared under normal living conditions and during a constant routine. However, when different rhythms are investigated, differences do exist, as described below.

##### The origin of the exogenous component

For example: the exogenous component is the sleep-wake cycle in the cases of heart rate and blood pressure, as with the rhythm of core temperature; it is the light-dark cycle for melatonin secretion; it is the rhythms of posture and fluid intake for urine flow; and it is the rhythm of food intake for plasma insulin concentration.

##### The relative size of the endogenous and exogenous components

As Figure 1 indicates, these two components are of similar size for core temperature. By contrast, the exogenous component is larger than the endogenous component in the cases of heart rate, blood pressure, urine flow and insulin secretion. For melatonin secretion, provided light levels are low (as normally found domestically) - the exogenous component is smaller than the endogenous component. Because the relative sizes of the endogenous components of the rhythms of core temperature and, particularly, melatonin are small, these rhythms are often used as 'clock markers'.

#### The body clock and its adjustment by zeitgebers

The body clock consists of paired suprachiasmatic nuclei at the base of the hypothalamus; many details of its genetics and molecular biochemistry are now known [4-6]. It is sited close to areas that exert widespread effects upon the body (for example, temperature regulation, hormone secretion and the feeding cycle) and sends outflows to these areas, so producing rhythmicity throughout the body.

When individuals are studied in time-free environments (such as an underground cave), the body clock and rhythms produced by it continue to be manifest but with a period closer to 25 than 24 hours. Such rhythms are called circadian (Latin: about a day) and the timing system is described as 'free-running'. This period of about 25 hours does not reflect the intrinsic period of the body clock (tau) exactly, due to effects of light exposure during waking. Current evidence (from protocols using very dim light during waking or from blind subjects) indicates that the true value of tau averages about 24.3 hours [7].

Whatever the exact value for tau in an individual, a body clock will be of value only if it and the rhythms it drives are synchronized to a solar (24-hour) day. This adjustment is due to zeitgebers (German: time-giver), rhythms in the environment and/or the individual's behavior which show a 24-hour period. For humans, the most important zeitgebers are the light-dark cycle and rhythmic secretion of pineal melatonin during the dark. Rhythms of physical activity, social factors and food intake appear to play comparatively minor roles [8-10]. Nevertheless, whatever the details of the components of a 'zeitgeber package', all potential zeitgebers normally act harmoniously to adjust the body clock to the 24-hour (solar) day.

The effect of light exposure upon the body clock depends upon the time of exposure relative to the temperature minimum (normally around 4 a.m. to 5 a.m., see Figure 1). Light exposure in the 6 hours after this minimum advances the body clock to an earlier time, in the 6 hours before the minimum, delays it, and at other times exerts no effect upon the clock [11]. Outdoor light is more effective at adjusting the body clock than is the dimmer light found indoors (that is, it tends to produce larger phase shifts), even though domestic lighting is normally adequate to act as a zeitgeber. The relationship between the time when a zeitgeber is presented and the shift of the body clock that is produced is called a phase-response curve.

Melatonin ingestion also adjusts the body clock; in the afternoon and early evening, melatonin ingestion advances the clock and, in the second half of sleep and during the early morning, delays it. Bright light inhibits endogenous melatonin secretion, and the clock-shifting effects of these two zeitgebers reinforce each other; bright light in the hours immediately after the temperature minimum advances the body clock directly via the phase-response curve (see above) and also indirectly (by suppressing melatonin secretion and so preventing the phase-delaying effect that melatonin would have exerted at this time) [12].

### Sleep rhythms

#### Laboratory-based results

A laboratory environment is advantageous in that conditions conducive to sleep (quiet, comfort, and so on) can be standardized. The standardization of such factors is equivalent to standardizing the exogenous components of the rhythms of sleep. Studies in sleep laboratories have systematically investigated the separate effects upon sleep and sleep architecture (the distribution of sleep stages) of time of day and time awake, which together constitute the endogenous component of the sleep rhythms. Several protocols have been used, including the constant routine, multiple sleep latency tests and modifying sleep-wake protocols (by changing the time of day while maintaining time awake constant or changing the amount of time awake at the same times of day). These protocols have been discussed in detail in Reilly and Waterhouse [13]. Some of the main findings from these investigations are detailed below.

Firstly, falling asleep is easiest if core temperature is falling or low (evening and night) and most difficult if it is high or rising (morning and afternoon). Secondly, waking up spontaneously tends to be the opposite of falling asleep; it is easiest if core temperature is rising or high, and most difficult when it is falling or low. These last results explain why, if individuals have gone to bed earlier or later than normal, even though they will feel less or more fatigued, respectively, than normal, they are likely to have a longer or shorter sleeps, respectively, than normal. Thirdly, the above two results stress that the ability to initiate and sustain sleep shows rhythmic changes during the course of the 24-hour day, and that these are associated with the rhythm of core temperature. However, superimposed upon these effects is that (if time-of-day effects are taken into account) sleep propensity - measured subjectively (fatigue, alertness) or objectively (polysomnography) - increases in proportion to time awake. This finding means that the endogenous component of sleep rhythms, like those of alertness, fatigue and cognition, is a mixture of two main elements, time of day and time awake. Finally, combining these findings indicates that an unbroken sleep is most likely to occur if it starts in the late evening and ends sometime after 7 a.m., the individual having slept through the trough of core temperature (see Figure 1). Sleep onset will occur as core temperature falls, melatonin secretion begins, fatigue increases and alertness falls, the individual having been awake for about 16 hours; sleep will end when core temperature rises and melatonin secretion falls.

#### Possible cause of rhythms of the sleep-wake cycle

The association between the sleep-wake cycle (sleep propensity, sleep maintenance and waking up) and the rhythm of core temperature has been interpreted as a causal link, incorporating temperature-induced changes in brain metabolism (see, for example, [14]). However, this might be an oversimplification of the position since there are many other rhythms associated with the sleep-wake rhythm [3,15]. These rhythms include: reciprocal activity of the sympathetic and parasympathetic branches of the autonomic nervous system (sympathetic activity paralleling core temperature and parasympathetic activity showing a profile that is the mirror image); levels of plasma adrenaline (parallel to core temperature); and plasma melatonin, concentrations of which are low in daytime light, start to rise around 9 p.m., peak during sleep in the dark, and fall on awakening in the morning. Melatonin is known to cause cutaneous vasodilatation (and so promote a fall of core temperature) as well as to increase fatigue. It is likely that many factors contribute to the physiological preparations that need to be accomplished to feel ready for sleep in the evening, to being able to maintain unbroken sleep at night, to making preparations for waking up in the morning, and to being physically and mentally active in the daytime. Core temperature is only one of these factors and should be seen as only one of the factors that reflect the activity of the body clock and rhythmicity of the body as a whole.

The value of the rhythms of core temperature and melatonin secretion as convenient markers of the timing of the body clock has already been mentioned. Alertness and fatigue, important indicators of sleep-mediated recuperation and the need for sleep, respectively, are also affected by the body clock. Once again, however, many other factors also contribute to these subjective feelings (for example, boredom and excitement with tasks in hand and, particularly, time awake). The effects of boredom and interest mean that alertness and fatigue can be misleading indicators as to the physiological need for sleep (the build-up of sleep pressure) at any time. Even so, the principles involved in modeling the rhythm of sleep times (see below) have also been used to model rhythms of alertness, fatigue and mental activity (for details of which, see [16-20]).

#### Sleep stages

Sleep is not homogeneous, and this has been investigated by recording surface electrical activity on the scalp using an electroencephalogram (EEG). In normal sleep, there is an ultradian rhythm of cycling between slow wave sleep (SWS) and rapid-eye-movement (REM) sleep stages, the REM-nonREM cycle. This cycle lasts approximately 90 minutes, about five cycles occurring during the course of a normal night's sleep. The composition of successive cycles varies, with the amount of SWS decreasing and the amount of REM sleep increasing.

### Modeling sleep rhythms and the distribution of sleep stages

#### The basic model

**Figure 2:**
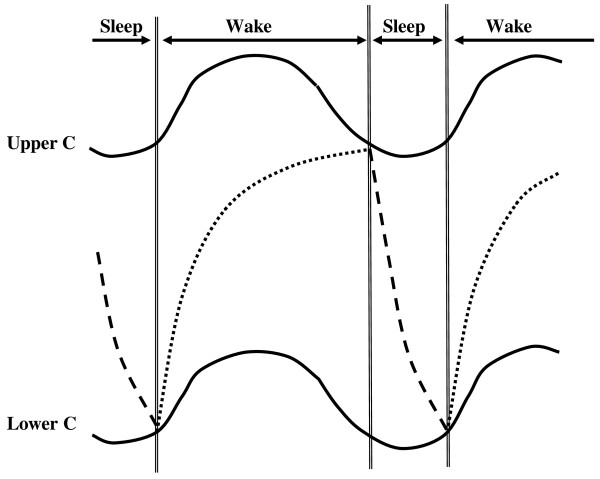
**Times of sleep and wake (top, separated by the double vertical lines)**. The C component is represented by two curves (Upper C and Lower C). Sleep pressure increases exponentially in the wake phase (Process S, dotted line) and decreases at a faster exponential rate in the sleep phase (S', dashed line). For more details, see text. (Based on [21]).

One simple, and yet very effective, model of sleep rhythms is the two-process model of sleep homeostasis of Borbély [21] (Figure 2). In this model, it is postulated that one of the processes, sleep pressure (S), increases during waking as an exponential saturating function, and then decreases exponentially during sleep (now being termed S'). The other process, C, is rhythmic with a period of about 24 hours and consists of two parallel components, an upper and lower component, both approximately in phase with core temperature.

Sleep onset occurs when the rising value of S intercepts the upper C function, normally during the phase of rapid decline and some hours before the minimum of the upper C function. During sleep, the level of S' falls exponentially (but with a shorter half-time constant than exists for S) until it meets the lower C function, at which point waking occurs and the cycle starts again. Waking normally occurs after about 7 hours of sleep, a few hours after the minimum of the lower C function when it is rising quickly. Factors S and C are assumed to act additively (but see [22,23]).

A three-component model of sleep regulation, based upon the two-component model but incorporating 'sleep inertia', has been developed. Sleep inertia is the phenomenon whereby, immediately after waking from sleep, the beneficial effects of sleep upon cognitive performance and mood are not immediately apparent [24-26].

#### Interpreting physiological and biochemical correlates of the sleep model

Many investigations have been performed to define the physiological and biochemical correlates of processes S and S' and the rhythmic function C, and others are in progress. As with investigations of sleep times, studies of processes S and S' generally consist of measuring aspects of waking or sleep (subjective estimates of fatigue and alertness, or objective measures of EEG during waking and sleep, for example) after the sleep-wake schedules of volunteers have been altered in some way.

The general view (summarized in [22,27]) is that the amount of sleep pressure accumulated during the wake time (process S) is reflected in the amount of SWS that occurs during the following sleep. Such 'deep sleep' has been regarded for some time as the type of sleep that best reflects its recuperative role. The actual dissipation of sleep pressure (process S') is associated with the amount of slow wave activity (SWA), assessed from the power in the low-frequency band of the sleep EEG. Increasing sleep pressure during the daytime when the subject is awake can be measured by assessing sleep propensity (the opposite of sleep latency) and the power in the theta-alpha band of the waking EEG. Sleep pressure is also related to increasing subjective fatigue and decreasing alertness. SWS and SWA are little affected by core temperature; by contrast, the amount of REM sleep is inversely proportional to core temperature and little affected by prior wake time. It is the difference between the factors associated with SWS and REM sleep that leads, during a normal nocturnal sleep, to the first REM-nonREM cycles being richer in SWS (beginning the process of recuperation) and the later ones, closer to the temperature trough, richer in REM sleep.

However, the detailed nature of the links involved remains unresolved. Comments already made about other variables being temporally associated with core temperature (and, therefore, the C component), and all of them possibly being reflections of some more fundamental process, apply here also. The fundamental reasons why increased wake time leads to an increased need for sleep and change in sleep architecture remain to be elucidated. One approach to this problem has been to investigate substances that accumulate in the brain during the waking period and the neurophysiological changes that take place during this time [28]. However, detailed explanations of the associations between sleep stages and time awake and core temperature are still awaited.

### Using the two-component model to explain altered sleep patterns in adults

#### Possible causes of differences in sleep patterns

With regard to adjustment of the body clock by zeitgebers, this process need not be precise on any particular day. Daily differences in detailed timing of biological rhythms will arise due to slight variations in the body clock and timing of the zeitgebers on a particular day. Moreover, different individuals will show biological variation with regard to the exact intrinsic period of their body clock (tau), their exposure to zeitgebers and details of their phase-response curves to different zeitgebers. That is, there will be both intra- and inter-individual variation in the timing of the circadian clock and the rhythms it drives.

Such variation will also apply when considering either the sleep homeostat (S and S') or rhythmic (C) components of the two-component model of sleep (Figure 2), all of which is predicted to lead to changes in the detailed timing of the sleep-wake cycle. Thus if the timing of the upper or lower components is changed, this will alter times of falling asleep or waking (times when the S and S' curves intercept the C curves); curves phased earlier will tend to cause earlier times of sleep onset and offset, and vice versa. If the amplitude of the C rhythms is decreased, the angle between the S and C curves will be less, and this will result in any changes in the curves causing increased variations in the exact timing of the points of intersection; this will in turn cause an increased variability in sleep onset and offset times. If process S increases less with time awake (its half-time constant is increased), then sleep onset will be delayed. Finally, if process S' is less marked (its half-time constant is increased), then sleep can be considered to be less recuperative (SWS and SWA will decline) and will tend to last longer.

These predicted changes can then form the basis of attempts to explain some abnormalities of sleep behavior, even though the understanding is incomplete in the absence of knowledge as to the exact correlates of the S, S' and C components (see above).

#### Adults who are poor sleepers and short-sleepers

Because, like any biological variable, the S, S' and C components will show inter- and intra-individual variation, there is the implication that the sleep-wake cycle will differ between individuals and between days in a single individual. It is common for individuals to delay their sleep-wake cycle over the weekend (generally due to increased social activities) and to find difficulty in readjusting it to weekday requirements on the Monday. There is also evidence that those with less regular lifestyles have more sleep problems [29]. These are external factors, and so contribute to the exogenous component of sleep rhythms, and readily modified by a change in lifestyle. The issue is if differences in the internal factors (S, S' and C) are present in some cases of altered sleep.

There are differences in the amount of sleep habitually taken by individuals because individuals choose to do this (external factors), seem to need less sleep ('short sleepers'), or get less sleep than they want and so feel tired in the daytime ('poor sleepers'). Studies using bed-rest and sleep deprivation have been used to investigate if poor sleepers show systematic differences from 'normal' sleepers [30], but they have not reliably shown that the sleep homeostat or rhythm of core temperature is different. For example, the temperature rhythm and responses to the multiple sleep latency tests following these changed schedules were normal in poor sleepers. Also, when short sleepers (< 6 hours per night) and long sleepers (> 9 hours per night) were compared, both responded in the same way with regard to the effect of prior wake time upon the amount of SWS and the kinetics of SWS during recovery sleep [31]. Therefore, it has been suggested that short sleepers endure higher sleep pressure, and that poor sleepers are not particularly susceptible to sleep pressure.

Even though these differences from normal sleep generally produce no more than some degree of inconvenience, they can be more troublesome if daytime fatigue is marked and cognitive function impaired. In these cases, treatment is sometimes contemplated, which generally consists of attempts to strengthen the individual's exposure to zeitgebers (particularly bright light) or regular ingestion of melatonin a few hours before sleep is desired [32-34]. In this regard, treatment is very similar to that for aged subjects (see below).

#### The effect of chronotype

The measurement of a person's chronotype score is now commonplace, the original questionnaire having been translated into several languages (and then validated upon local populations of subjects) and also adapted for cultures that, for example, routinely rise early. A population's chronotype scores are distributed normally, with those who tend towards the 'morningness' part of the distribution choosing to perform important activities in the morning, and those tending towards 'eveningness' choosing to do them in the evening. The tails of the distribution - the extreme morning types (larks) and extreme evening types (owls) - can find normal lifestyles difficult to participate in fully and effectively. This difference might be due to habits (and so influence the times of exposure to zeitgebers) and/or the body clock (tau values differing between individuals) and/or to the phase response curves (which might differ in the size of advances and delays in the body clock that zeitgebers produce), but few data on this issue are available. What can be stated is that individuals are likely to have problems if they adopt an early lifestyle (retiring and rising earlier than average) but have a circadian system that tends to run later than average (for whatever reason), or vice versa. Such disparities can be important for those working shift systems.

The observation [35] that temperature rhythms in morning- and evening-types during constant routines are phased about 1 hour earlier or later than average, respectively, implies that some endogenous component (differences in adjustment of the body clock? differences in tau?) is involved, but it might also be caused partly by the phase differences (due to different lifestyles) that were present in the days before the constant routine.

The S and C components of the two-process sleep model have been compared in morning and evening types, using the core temperature rhythm (a reflection of the C component) and daytime measures of subjective sleepiness and alpha-theta activity (reflections of the S component). The core temperature was phased earlier, and the build-up of sleepiness and alpha-theta activity was more rapid, in the morning types; that is, both components of the two-component model were different [36]. In other studies, morning-types had more SWA at the start of a recovery sleep following a night of sleep disruption, and also showed a more rapid decay of SWA [37,38]. These results indicate that both processes S and S' might be more rapid in morning types, in addition to an earlier phasing of the body clock (see above), and all factors might contribute to earlier times of retiring and rising.

There is evidence that some of the differences between chronotypes and in processes S, S' and C have a genetic basis. Evidence from a large study upon adults in which sleepiness, chronotype, quality of life and sleep times were compared [39] indicated that associations existed that could not be explained by common lifestyles (those sharing the same household, for example); this implies a genetic component. One of the clock genes, PERIOD3, shows polymorphism and individuals homozygous for one variant, the five-repeat allele, PER3(5/5), are not only more likely to be a morning type but also more susceptible to the effects of sleep loss. By contrast, individuals homozygous for another variant, PER3(4/4), are more likely to be an evening type. Subjects homozygous for PER3(5/5) also showed more waking alpha-theta activity, more SWA during sleep and a greater decline in cognitive function when sleep-deprived [40]. A previous study [41] had also shown that individuals could be divided into 'resistant' and 'non-resistant' on the basis of deterioration of their brain function following sleep loss; since this division was independent of any difference in phasing of core temperature, it seems to relate to the sleep homeostat rather than process C.

Two comparatively rare syndromes have been described: delayed sleep phase syndrome and advanced sleep phase syndrome. In the former, the internal relationship between daily rhythms and the sleep-wake cycle is normal, except that the whole system is delayed with regard to external time; for example, the core temperature rhythm shows a minimum around 8 a.m., and melatonin secretion begins around midnight (both rhythms being delayed about 4 hours compared with normal). Subjects tend to wish to retire around 4 a.m. and rise about noon, and this lifestyle can be very inconvenient [42]. The opposite changes in timing of daily rhythms and chosen sleep-wake cycle apply to advanced sleep phase syndrome. Behaviorally, the individuals can be considered as pathologically extreme examples of evening or morning types. Whether the syndromes result from extreme values of tau, abnormal phase-response curves, and/or abnormalities with regard to the sleep homeostat is unknown.

In summary, evidence is beginning to be obtained that indicates individuals' chronotypes partly reflect some aspects of the timing of their body clock and sleep homeostat, and that a genetic component contributes to these differences.

### Differences in sleep patterns with age

#### Neonates and infants up to adolescence

Immediately after birth, neither full-term nor premature babies show clear circadian rhythms of the sleep-wake cycle or any other variable [43-45]. Instead, bouts of sleep and waking alternate several times during the course of a 24-hour period. Therefore, the concept of sleep pressure, accumulating with substantial periods of waking, cannot be applied in the same way. Further, EEG results indicate that the nature of sleep is different, there being two types of sleep, 'quiet' and 'active' [46-48]. That is, the basic two-component model of sleep cannot be applied to individuals in newborn babies.

Even if the sleep-wake rhythm and other circadian rhythms developed progressively during the first years of life, to become firmly established when the child is five years of age [49-52], there are no studies in which components of the two-component model have been investigated in children of this age. The reasons for this are easy to understand - requiring infants to take part in such studies would be unethical.

#### Adolescents

In practice, therefore, the earliest age at which substantial amounts of data have been collected is when the child reaches adolescence, though here also experimental sleep-deprivation experiments have not been performed and no constant routine data are available. The data that are available for this age group (10 to 17 years) are dominated by the observation that they go to bed considerably later in the evening (particularly if they have access to television or live in latitudes where summer evenings can be very long) and so tend to be sleep-deprived on school days [53-64]. At weekends, they catch up on lost sleep by extended time spent in bed (lie-ins). At the weekends also, the melatonin rhythm is phase delayed compared with during the week. Adolescents tend towards evening types, therefore, though this change is more marked in some than others and occurs at a slightly earlier age in girls, possibly due to their earlier onset of puberty [65]. The sleep restriction that is experienced during weekdays causes concern with regard to school performance, particularly when school starts early in the morning and the degree of eveningness of an individual is marked [66-71].

**Figure 3:**
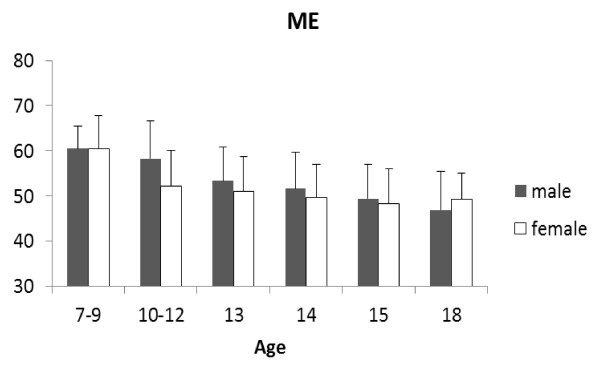
**Morningness-Eveningness Questionnaire (chronotype) scores in male and female school children of different ages**. Filled bars, boys; open bars, girls. Scores indicate: range 16 to 30, definitely evening-type; 31 to41, moderately evening-type; 42 to 58, neither or intermediate-type; 59 to 69, moderately morning-type; and 70 to 86, definitely morning-type.

Some of these points are illustrated by some of our unpublished data, which show changes in chronotype and sleep quality in primary and secondary school students in Japan (7 to 18 years old; 404 boys, 411 girls). The Japanese versions of Horne and Ostberg's Morningness-Eveningness Questionnaire (MEQ) and the Pittsburgh Sleep Quality Index (PSQI) were used. Figure 3 shows the MEQ scores. Although most of the students were neither morning type nor evening type, that is, they were 'intermediate type', the scores decreased significantly with age, indicating a change towards becoming evening type (boys: r = -0.99, *P *< 0.001; girls: r = -0.83, *P *= 0.041, Pearson's correlation coefficients). Female students older than 10 years tended to be more evening types than their male counterparts, but this difference decreased with age and was reversed in students aged18 years old. There was a clear linear relationship between the MEQ scores and age of male students, while this relationship was less clear in the female students and appeared to be affected other factors, especially around 10 years of age. This change in female MEQ scores at around 10 years might relate to menarche.

**Figure 4:**
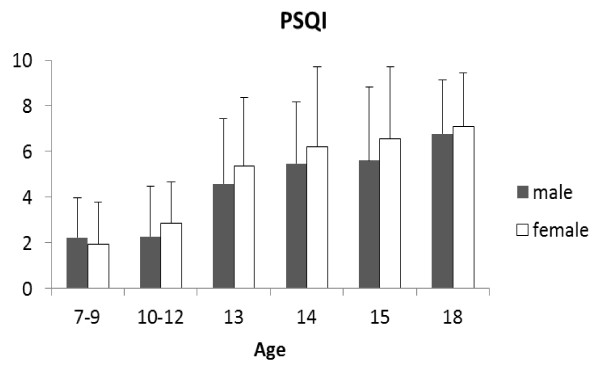
**Pittsburgh Sleep Quality Index scores in male and female school children of different ages**. Filled bars, boys; open bars, girls. Scores > 5 indicate some problems with sleep.

Figure 4 shows that their PSQI scores tended to increase significantly with age, indicating a deterioration in sleep quality (boys: r = 0.96, *P *< 0.01; girls: r = 0.95, *P *< 0.01, Pearson's correlation coefficients), girls tending to be worse than boys. Given that all students had to rise at the same time in the morning to attend school, the poorer sleep observed in the older children was consistent with the tendency for the chronotype in older children to become progressively more orientated towards that of an evening type.

Attempts have been made to incorporate the findings regarding lifestyle and chronotype in adolescents into the two-component model of sleep regulation [72-74]. It is believed that both components change. The evidence for changes in the C component is the later phasing of core temperature and melatonin rhythms; the evidence for a change in the sleep homeostat (S and S' components) is an increase in sleep latency and a decline in delta power density. From a circadian viewpoint, the delayed rhythms imply that early school hours will be too soon after the trough of the core temperature rhythm and time of high melatonin secretion (resulting in poorer cognitive performance), and the fall in core temperature and onset of melatonin secretion later in the evening will delay sleep onset. The observed changes in the sleep homeostat appear to enable these later bedtimes to occur.

Why such changes should exist during and just after puberty is unclear; attributing them to the marked maturational changes, in the brain as well as the pituitary-gonadal axis, is an obvious possibility, but details of the mechanisms involved are unknown [75,76]. It is also possible that the individual's increasing independence at this age means that personal interests might delay bedtime, and this will lead to a delay in the circadian system. If this exogenous factor were important, then curtailing evening activities and retiring earlier would provide a suitable remedy; but if the driving force behind the change were neurologically or hormonally-based (and internally-based), then advising earlier retiring times would be ineffective. Whatever the cause, recommendations that school hours and examination times should be delayed are common [66,68,70,71].

#### Older persons

There has been a large amount of research upon the sleep-wake cycle and associated rhythms in aged individuals, generally 55 years or older (for recent reviews, see [77-79]. Volunteers for such investigations have come from two main sources; those who come to the laboratory and those who live in homes for the elderly. The type of person studied in these two cases might not be the same. Subjects volunteering for laboratory-based studies are likely to be healthy, active and independent; those studied in homes are more likely to be less active and independent, and also more likely to suffer from some of the problems associated with old age - forms of dementia, for example. The concepts of 'survivor' and 'frailty', respectively, have sometimes been used to describe these two types of volunteer [80], and differences between the results given by them might be expected.

Nevertheless, there are the following general findings [58,81-88]: daytime is associated with a greater number of naps and night with greater sleep latency and more fractionated sleep (sleep efficiency declines); the increased frequency of waking is associated with a need to empty the bladder; and times of retiring and rising tend to become slightly earlier, individuals become more of a morning type. These changes to sleep and the sleep-wake cycle have been observed in longitudinal [89,90] as well as transverse studies, and made use of measurements that have come from self-report diaries, answering questionnaires, reports of care-givers or objective measures of activity (by actimetry) or sleep (EEG and polysomnography).

Other factors that might be involved with changed sleep-wake cycles are also found in aged individuals. Poorer thermoregulation [91] and cognitive function [92] are present but, as in younger adults, a nap improves cognitive performance [93]. Poorer sleep at night and increased daytime naps will decrease the normal degree of dichotomy between daytime activity and nocturnal rest. Associated with this decrease is likely to be decreased regularity of lifestyle and smaller exogenous components of circadian rhythms. Against this scenario, however, are the results from studies that have shown an increase in lifestyle regularity [94-96]. Also, following a simulated time-zone transition, aged persons, like younger adults, suffer from sleep loss and deterioration in cognitive performance - but older subjects seem better able to pace themselves and maintain cognitive performance at times of sleep loss and low core temperature [97,98]. There is also other evidence that alertness in the aged seems to be less dependent upon core temperature than is the case in younger subjects [99]. In other words, some of the behavioral changes observed in aged subjects accord with the view that individuals try to adopt lifestyles that oppose the difficulties that arise with aging.

Circadian rhythms alter with aging; those of core temperature and melatonin secretion having been studied most. The amplitudes of these rhythms, when measured in aged individuals living their normal lifestyles, normally show lower values when compared with younger controls and phase advances in the order of 1 hour [85,100]. There is also evidence that aged individuals who suffer from poor sleep at night have a raised core temperature at this time [101], but this is in contrast to the results from an earlier study that had found that altered sleep could not be accounted for by changed rhythms of core temperature [102]. Such deterioration in circadian rhythms is not observed equally in all aging subjects [103,104] but tends to be more marked when other problems (such as pain, failing cognitive powers or senile dementia) are also present, and less marked if a partner or companion is present.

Determining the cause of the observed changes is difficult because the links that exist between circadian rhythms and the sleep-wake cycle are complex. For example, the need for recuperation during nocturnal sleep might be lessened if, during the daytime, an individual is less physically and mentally active and takes naps. Also, even though decreased activity of the suprachiasmatic nucleus (producing a rhythmic output that is lower in amplitude) could account for many of the observed changes, so too could, for example, decreased exposure to or perception of zeitgebers, daytime activity and exposure to the natural light-dark cycle being compromised by decreased mobility (not being able to venture outdoors) and/or poorer eyesight.

Poorer vision might be particularly important, causing individuals to lack confidence and further restrict their daytime activities, both indoors (reading, for example) and outdoors (walking). The cornea and lens become more opaque with age and light transmission, particularly of the shorter (bluer) wavelengths, decreases [105]. This acquired tritanomaly can be assessed objectively by a desaturated 15-hue test, in which patients are required to distinguish between very similar shades of blue. This partial loss of perception of blue colors can also be seen in some paintings in which, as the artist gets older, there is more interest in reds and yellows than greens or blues. These changes in the perception of blue hues can be marked in those who suffer from cataracts.

Light of shorter wavelengths is also important in the control of body temperature, melatonin secretion and the sleep-wake rhythm. Melatonin secretion in the late evening and night promotes sleep, partly due to the fall in core temperature that it produces. This secretion is suppressed by light, particularly light at the blue end of the visible spectrum [106-108]. Accordingly, it seems possible that changing transmission of blue light through the lens in aged subjects, particularly those suffering from cataracts, will change the pattern of melatonin secretion and times of sleep. For example, the declining transmission of blue wavelengths in the evening might lead to an earlier rise of melatonin secretion in the evening, contributing to earlier sleep onset in aged individuals. In addition, it has been observed [109] that daytime exposure to bright light in young adults raises the secretion of melatonin at night, causes a greater fall of core temperature at this time, and improves sleep; such effects might be less marked if the lens of the eye has reduced ability to transmit light at some, or all, of the visible wavelengths.

Therefore, cataract surgery will not only improve individuals' sight but it might also alter the quality of their sleep. Asplund and Lindblad [110,111] found that a considerable proportion of the patients who had undergone cataract surgery reported subjective improvements in their sleep after surgery. This has recently been re-examined [112]. Fifteen patients were studied before and one month after cataract surgery, in which UV light-cutting intra-ocular lenses had been implanted. Vision and color perception both improved. After recovery from the surgery, they also demonstrated a change in nocturnal sleep, though this improvement varied between patients. After surgery, patients showed a negative correlation between later wake-up or retiring times and sleep efficiency; that is, sleep efficiency of patients with earlier wake-up and retiring times was higher than in those with later wake-up and retiring times. It seems that, after surgery, the patients' retinas received more light of shorter wavelengths and this affected sleep efficiency.

Whilst the detailed implications of these results with regard to the link between light perception and sleep are still unclear, they do confirm that light, melatonin secretion and sleep are linked. It is also noteworthy that, in aged individuals who do not suffer visual problems, the phase shifts produced by exposure to bright light are the same as in younger controls [113]. Also, a 3-hour bout of exercise at the start of normal sleep time delayed the melatonin rhythm equally in young and old adults [114]. That is, there is no evidence that the increased morningness found in aged individuals can be attributed to altered adjustment of the body clock by zeitgebers.

Attempts to interpret the causes of altered sleep patterns in the aged in terms of the two-component model of sleep provide some evidence that both the C and S components are altered, but interpretation of such results is not without problems. For example, whilst many circadian rhythms show decreased amplitude, this decline can be due to a fall in the endogenous or the exogenous component of the rhythm, or both. That is, the observed fall in amplitude of the rhythm of core temperature (which implies a change in the C component) might reflect decreased output from the suprachiasmatic nucleus (the endogenous component of the rhythm), decreased secretion of melatonin (which causes core temperature to fall due to cutaneous dilatation), or a decrease in the dichotomy between daytime activity and nocturnal inactivity (the exogenous component). Further, the tendency for aged individuals to become more morning-orientated might be due to an altered body clock and/or timing of individuals' lifestyles (factors that might reflect the C component of the two-component model in particular), or the more rapid build-up of sleep pressure (the S component). A further difficulty of interpretation arises because older individuals might go to bed earlier because their body clock is running faster, their poorer eyesight restricts what they can do (read or watch television), their declining mental faculties mean they get bored more easily, sleep pressure builds up more quickly or they fear a poor sleep at night and so want to attempt it sooner.

It is only the endogenous component of the rhythm of core temperature that is directly associated with component C. Since the endogenous and exogenous components of a circadian rhythm normally act together to produce a measured rhythm, it is necessary to distinguish between them. The constant routine protocol is believed to enable the circadian output from the body clock to be assessed more directly. However, it must be remembered that this protocol assumes that any effects of the sleep-wake cycle upon the amplitude and phase of the core temperature rhythm on normal days (when the exogenous component is present) do not continue into the constant routine but disappear immediately it is undertaken. There is some evidence to support this assumption, at least with regard to amplitude [115]. However, when aged participants undergo constant routines and their core temperature rhythm or secretion of melatonin in dim light is investigated (two common markers of the body clock), there is evidence for a reduced output from the body clock insofar as the amplitude of these rhythms is reduced; also, the temperature profile has been found to advance [116,117]. These results suggest the presence of a body clock with a declining output and an advanced timing. However, when the free-running period of the body clock was examined in aged individuals who were blind (twice in each individual, the occasions of study being separated by a period of 10 years), where effects of light upon tau are absent, the period lengthened [118]. Other studies also have failed to find a consistent phase advance of the core temperature rhythm in sighted individuals when using the constant routine protocol. Only an advance of phase of the core temperature rhythm, possibly due to a reduction in tau, would indicate an endogenous cause for the increasing tendency to morningness with aging (rather than an exogenous cause such as preferring to get up earlier and go to bed earlier). Therefore, the general position with regard to the change in the endogenous component of the temperature rhythm in aged individuals is unclear. Again, differences between survivors and the frail might be important.

The changes in sleep with aging might be explained in terms of altered S and S' processes. Investigations of responses to altered sleep-wake schedules (advancing or delaying bedtime, for example) have shown that changes similar to those found in younger adults under the same circumstances are found for many sleep variables, including sleep efficiency and time awake after sleep onset [119]. The increase in sleep latency with age has been taken to imply that sleep pressure decreases but, again, contradictory results have been obtained. SWS during the first part of a night's sleep is normally decreased in the aged [120,121]. The rate of dissipation of S (S', as assessed from SWA) does not differ from that found in younger adults and, as in them, the amount of SWS increases following sleep-deprivation [122] and decreases in nocturnal sleeps taken after daytime naps [123]. On the other hand, another study claimed that the relationship between SWS and prior wakefulness was different between aged participants and younger controls [116] and that daytime naps led to a fall in sleep efficiency and earlier waking [124]. Several differences in the power of different frequency bands in the EEG have been found in aged individuals compared with younger controls [120], though their detailed meaning is unclear.

It has already been mentioned that those who seem to experience fewer difficulties with sleep also seem to have more regular lifestyles. It is stressed that this increased regularity might reflect a relatively stronger output from the body clock (process C) or stronger activity of the sleep homeostat (processes S and S') - or it might be that increasing the exogenous components of circadian rhythms, attempting to be active and so promote accumulation of sleep pressure when awake, and having regular exposure to zeitgebers, can all be considered as ways of combating a declining influence of endogenous components. Whatever the exact cause of the decreased daily variability in some aged individuals, it becomes translated into several ways of improving the quality of life of the aged, at least in those living in homes for the elderly [125].

The general aim of these procedures is to increase the dichotomy between daytime activities in the light and nocturnal sleep in the dark [1,126-137]. Possibilities include increasing daytime physical activity, outdoors in natural light, if possible and increasing daytime mental activities, by providing an interest or getting individuals to discuss topics of interest to them (their childhood, favorite food or films, for example). Increasing artificial lighting levels is also used, not only to promote conditions suitable for taking part in activities but also to inhibit napping. Coupled with these courses of action can be encouragement to individuals to stay in bed at night, even if they cannot sleep, with restricted use of lights (as long as requirements for safety and care are met). In addition, some studies have indicated that the regular use of a mild soporific is effective (melatonin, for example), though the long-term use of any drug requires medical advice [135,138,139].

Many of these treatments have been found to be effective; it often being stressed that individuals should be encouraged to see some form of regular daytime activity as a simple means of improving their quality of life. The possible mechanisms which might cause the treatments to be effective have been considered above; they might act on the exogenous or endogenous component of circadian rhythms by promoting accumulation of sleep pressure, and promote sleep pressure directly. Which mechanism(s) are important is not yet known.

A rather different approach to the problem of improving sleep in older individuals is based upon the decline in efficiency of thermoregulation with age [91]. Part of the problem is that, due to impaired cutaneous thermal sensitivity, aged individuals are less able to perceive the temperature of their surroundings, and so are unable to forestall falls or rises of body temperature by suitable behavior. Additionally, sleep onset is inhibited by cold hands and feet, and this is observed in all age groups. Warming the feet by wearing bed-socks was found to decrease sleep latency in aged individuals [140].

## Conclusions

The two-process model of sleep has enabled the processes that determine sleep times and some aspects of sleep architecture to be described. Equally, the constant routine protocol remains the standard way of distinguishing between endogenous and exogenous components of circadian rhythms. Taken together, these concepts of a sleep homeostat and endogenous and exogenous components of circadian rhythms have enabled the quantity, timing and quality of sleep to be understood better. Such understanding has been applied successfully to understanding the timing of sleep in healthy individuals and in the changed circumstances of adolescence and old age. However, details of the nature of the S, S' and C components of the sleep model, and unraveling the interactions between these components and those of the circadian rhythms, are still required. Obtaining such details will improve our knowledge of differences between healthy individuals at different stages of their life-span and when suffering from some sleep disorders. Such deeper understanding will then give a firmer rationale to advice and treatment, with the hope that they will become more successful.

## Competing interests

The authors declare that they have no competing interests.

## Authors' contributions

The preliminary draft of part of the section on adolescents was written by YF, of part of the section on old persons by TM, and of the rest of the review by JW. Thereafter, all authors were equally involved in the several revisions that were undertaken. All authors read and approved the final manuscript.
